# Struggling to Keep Up and Have a Good Life: A Qualitative Study of Living With Impaired Balance Control Due to Multiple Sclerosis

**DOI:** 10.1093/ptj/pzad065

**Published:** 2023-06-20

**Authors:** Andreas Wallin, Erika Franzén, Urban Ekman, Sverker Johansson

**Affiliations:** Division of Physiotherapy, Department of Neurobiology, Care Sciences and Society, Karolinska Institutet, Huddinge, Sweden; Aleris Rehab Station Stockholm, Research and Development Unit, Stockholm, Sweden; Division of Physiotherapy, Department of Neurobiology, Care Sciences and Society, Karolinska Institutet, Huddinge, Sweden; Women’s Health and Allied Health Professionals Theme, Medical Unit Occupational Therapy and Physiotherapy, Karolinska University Hospital, Stockholm, Sweden; Stockholm Sjukhem Foundation, Research and Development Unit, Stockholm, Sweden; Division of Clinical Geriatrics, Department of Neurobiology, Care Sciences and Society, Karolinska Institutet, Stockholm, Sweden; Women’s Health and Allied Health Professionals Theme, Medical Unit Medical Psychology, Karolinska University Hospital, Stockholm, Sweden; Division of Physiotherapy, Department of Neurobiology, Care Sciences and Society, Karolinska Institutet, Huddinge, Sweden; Women’s Health and Allied Health Professionals Theme, Medical Unit Occupational Therapy and Physiotherapy, Karolinska University Hospital, Stockholm, Sweden

**Keywords:** Balance Control, Balance Impairment, Content Analysis, Multiple Sclerosis, Qualitative Research

## Abstract

**Objective:**

We aimed to explore and describe the experiences of people with multiple sclerosis (MS) living with impaired balance control and how balance impairment can be managed in everyday life.

**Methods:**

A qualitative design was used**.** Data were collected through semistructured interviews. Transcripts were analyzed using qualitative inductive content analysis. Sixteen participants (12 women) with MS and variation in level of balance control were interviewed**.** Age ranged between 35 and 64 years, and overall MS-disability ranged between 2.0 (mild) and 5.5 (moderate) according to the Expanded Disability Status Scale.

**Results:**

Five main categories emerged: Balance is an automatic skill that now requires attention; contributors to balance impairment; burdens of balance impairment; management of balance impairment; and negotiation between capacity and ambition for continuing the good life. Body functions emphasized as central to keeping balance were somatosensory-motor functions, vision, and management of fatigue. Day-to-day variation in capacity and being in stimuli-rich environments were conditions highlighted as impacting balance. The main categories yielded the overarching theme of being restrained by impaired balance control and struggling to keep up.

**Conclusion:**

Participants with MS described balance impairment as balance no longer being an automatic skill and having an adverse impact on everyday life. A strong effort was shown to not let shortcomings control and determine quality of life. To manage limitations and restrictions and to move forward in the struggle to keep up a good life, an extensive toolbox of strategies aiming to minimize the impact of balance impairment was used to maintain quality of life.

**Impact:**

This study highlights the importance of person-centered health care in MS, with increased awareness of the individual perspective of how balance impairment is perceived. The person-centered focus increases both quality and efficiency in therapy since it involves the individual’s thoughts of a life where participation in valued activities is less restricted.

## Introduction

Multiple sclerosis (MS) typically debuts in young adulthood, and most of those affected are women.^1^^,^^2^ Balance control derives from the interactions between sensory, motor, and cognitive functions,^3^ which are commonly affected in people with MS (PwMS).^4^^,^^5^ Balance impairment often occurs also in the early stages of the disease,^6^ even in PwMS with minimal or no clinical signs of other impaired body functions associated with MS.^7^ Due to balance impairment, falls and fear of falling are common,^8^^,^^9^ contributing to activity curtailment and participation restriction,^10^ and influencing health-related quality of life.^11^

Quantitative research in PwMS has explored interventions aiming to improve balance control and reduce falls,^12^ with promising results.^13–15^ In qualitative research in PwMS, falls and the perceived causes of falling,^16^^,^^17^ and how the effects of balance training interventions are perceived and facilitate activity^18^^,^^19^ have been described. Yet, to improve our knowledge of how such interventions shall be delivered, PwMS’ perspectives of how balance impairment impacts their lives need to be better understood. Furthermore, PwMS have described how embarrassment related to one’s balance impairment and perceptions of others’ attitudes are barriers to continue being physically active.^20^ A more comprehensive picture of how impaired balance control impacts participation in activities that give meaning and are valued in PwMS’ everyday life is still lacking. Thus, the objective of this study was to explore and describe PwMS’ experiences of living with impaired balance control, how it impacts activity, and how it can be managed in everyday life.

## Methods

### Design and Participants

This study used a qualitative design with an inductive approach. Participants were strategically selected from a study cohort originally recruited for a reliability study.^21^ Participants in the reliability study were recruited from MS specialist centers and clinical rehabilitation units in Stockholm, Sweden. Inclusion criteria were PwMS diagnosed according to the McDonald criteria,^22^ with an overall MS-disability score of 2.0 to 5.5 according to the Expanded Disability Status Scale,^23^ aged from 18 to 65 years, and able to walk 100 meters without aid. Exclusion criteria were cognitive impairment as indicated by a score < 21 in the Montreal Cognitive Assessment,^24^ presence of other conditions that would substantially influence balance, an MS relapse, or change of disease-modifying treatment in the 8 weeks prior to sampling, alcoholism, or pregnancy.

Of the 54 PwMS included in the reliability study, 16 were invited and agreed to participate in the qualitative study. The strategic selection was based on relevance sampling,^25^ with selection by optimized variation in overall MS disability, sex, age, and level of balance control based on the balance measure, Mini-BESTest.^25^^,^^26^ Sufficiency in spread and variation within the sample regarding the aforementioned parameters, along with the coverage of research questions’ answers, determined the number of participants interviewed. Of the 16 participants interviewed, 12 were women. Ages ranged from 35 to 64 years, and overall MS-disability ranged from 2.0 to 5.5. Three participants used walking aids inside and outdoors, 8 used them only when outdoors. Participant characteristics are presented in Table 1. All participants signed the informed consent. Procedures were conducted in accordance with the Declaration of Helsinki. The ethical review board in Stockholm approved the study, Nos. 2018/374-31 and 2019-01562.

**Table 1:** Characteristics of Participants With Identification P1 to P16*^a^*

Participant ID	Sex	Age Range, y	Time Since Diagnosis, y	Clinical Course	Overall MS-Disability (EDSS)	Mini-BESTest Score	Have Fallen in the Last 6 Months	Use of Walking Aid
P1	Female	55–64	19	Progressive	4.0	22	Yes	None
P2	Female	45–54	10	Relapsing remitting	5.0	15	No	In- and outdoors
P3	Male	35–44	6	Progressive	5.5	22	No	In- and outdoors
P4	Male	35–44	0	Relapsing remitting	2.0	21	Yes	Outdoor
P5	Male	45–54	5	Relapsing remitting	3.5	18	Yes	Outdoor
P6	Female	45–54	20	Relapsing remitting	5.0	14	Yes	Outdoor
P7	Female	55–64	23	Progressive	3.5	22	No	None
P8	Female	55–64	16	Relapsing remitting	3.0	12	Yes	Outdoor
P9	Female	45–54	29	Relapsing remitting	4.0	8	Yes	None
P10	Female	55–64	2	Relapsing remitting	4.5	17	Yes	Outdoor
P11	Female	55–64	13	Relapsing remitting	5.5	21	No	Outdoor
P12	Female	45–54	22	Relapsing remitting	4.0	20	No	None
P13	Female	55–64	9	Relapsing remitting	4.0	20	No	None
P14	Male	55–64	3	Progressive	4.5	19	Yes	Outdoor
P15	Female	45–54	19	Relapsing remitting	4.0	15	Yes	Outdoor
P16	Female	45–54	19	Relapsing remitting	5.0	16	Yes	In- and outdoors

^a^ EDSS = Expanded Disability Status Scale; ID = Identification.

### Data Collection

**Figure 1:**
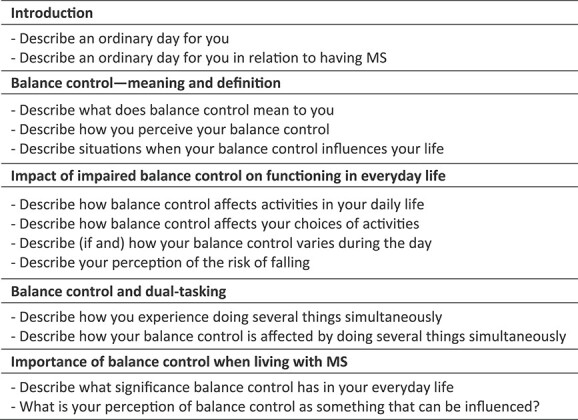
The interview guide.

Demographic data were collected during the reliability study assessment. Qualitative data were collected at least 2 months later through semistructured interviews conducted by the last author, who was not involved in the demographic data collection. Interviews were conducted at Karolinska Institutet, in the participants’ home or via video link. They were audio-recorded using a Dictaphone (Olympus VN-741PC). Data were collected from August 2019 to June 2020; the average interview length was 60 minutes. A study-specific interview guide with open-ended questions was used (Fig. 1). Question areas were: balance control—meaning and definition; impact of impaired balance control on functioning in everyday life; balance control and dual-tasking; and importance of balance control when living with MS. The first 2 interviews were discussed between the first and the last author to evaluate the interview guide, which resulted in minor guide adjustments for the following interviews.

### Analysis

The audio-recorded interviews were listened to and transcribed verbatim. Transcripts were analyzed using qualitative content analysis,^25^ according to the inductive process described by Elo and Kyngäs.^27^ First, transcripts were read in their entirety by the authors A.W. and S.J. Notes and headings were written down to describe all aspects of the content. They were then discussed to achieve consensus on what was significant in relation to the study objective and to ensure conformability. From the notes and headings, subcategories were generated and then grouped into generic categories and main categories. Content-characteristic words were used to label each main category. Iteratively, the authors returned to the original transcribed interviews to enhance analysis trustworthiness and credibility. The relationship between main categories was discussed thoroughly by the first and the last authors. Afterwards the results were discussed with co-authors E.F. and U.E. until consensus on an interpretation of the results was reached. An overarching theme was then generated. Table 2 exemplifies the initial analysis stages. Quotations are referred to by the participant ID, presented in bold font as in Table 1.

**Table 2:** Example of the Initial Stages of the Analysis Process From Generating Notes and Headings to Creating Codes and Subcategories

Participant ID	Note or Heading	Code	Subcategory
P2	My energy runs out and I kind of collapse, like a folding knife	Fatigue impact—physical	Energy-related internal factors that affect balance control
P16	I do not know when my leg may suddenly buckle, if I am standing and doing something	Not trusting your legs gives uncertainty of ability	Living with the risk of falling
P12	It’s not such a fun life. It’s pretty boring...Simply, my fatigue affects me	Impact of fatigue	Loss and limitations in activity affects quality of life
P10	My legs don’t move at the same pace as I do…My legs have a slow pace, and I’m often ahead of them…I think I’m walking, but my legs aren’t	Description of changes in coordination and agility	Changes in functioning
P9	I have to relate to it and accept it. Because otherwise I’ll become a victim, with self-pity, and that’s not who I am	Management of capacity	Acceptance of disability
P14	I still have a good life	Approach to one’s situation	Mindset for keeping quality of life

#### Role of the Funding Source

The funders played no role in the design, conduct, or reporting of this study.

## Results

**Figure 2:**
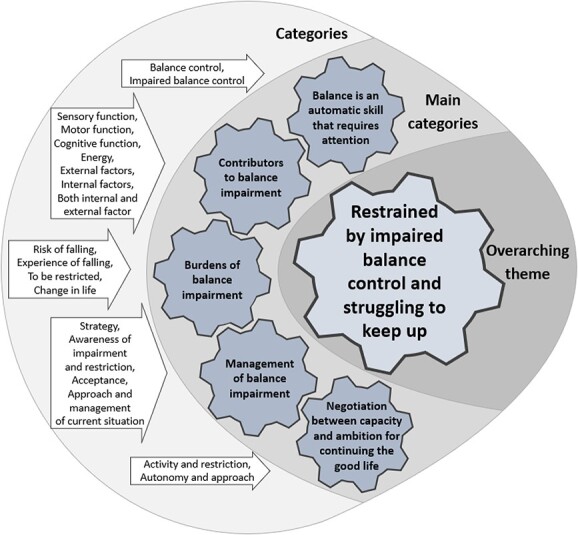
The figure depicts the categories, main categories, and the overarching theme generated during data analysis.

Five main categories emerged in the analysis describing how impaired balance control was perceived, experienced, and managed in everyday life among PwMS. Interpretation of the essence of the main categories yielded the overarching theme “restrained by impaired balance control and struggling to keep up”. Figure 2 depicts categories, main categories, and the overarching theme generated during analysis.

### Balance Is an Automatic Skill That Now Requires Attention

Unaffected balance was perceived by participants as a basic, automatic skill enabling the individual to move around freely without the risk of falling and without requiring concentration, as 1 participant described: *“Balance entails being able to physically move around without needing to think”* (P2). Being continuously capable, without attention to adapt and manage your balance in most activities, was perceived as normal. However, attention to one’s balance could still be a prerequisite for moving around with self-confidence in more advanced activities, such as walking on a narrow footbridge.

On the contrary, maintaining balance when it was impaired due to MS required increased attention and concentration: *“Worsened balance means that I need to be aware when I’m walking around outdoors”* (P14). The margin for what required awareness to keep one’s balance had become lower; attention was needed even to perform movements that, before disabilities appeared, were considered simple, unchallenging, and did not threaten self-confidence: “*The bar for what requires balance and challenges balance has been lowered. Before, balance that requires awareness represented something advanced, but now it’s about being able to put on shoes without falling over*” (P15). The experience of balance impairment contributed for some to perceptions of a distance between self and body, that the body did not move automatically, as it was supposed to do: “*My legs don’t move at the same pace as I do…My legs have a slow pace, and I’m often ahead of them…I think I’m walking, but my legs aren’t”* (P10).

### Contributors to Balance Impairment

Impairments in various body functions were the primary contributors to how and why participants’ current balance control was affected, either solely or in interaction with other functions. For some, it was evident that for maintained balance control, substitute body functions were used to compensate for impaired ones.

Impaired somatosensory function caused unsteadiness and uncertainty: *“I have somatosensory impairment everywhere, including under my feet, and that makes everything a little more insecure”* (P6). Impaired motor strength, explosivity, and/or agility also affected their balance control, making moving around unreliable and unpredictable when 1 was unaccustomed to how their body now behaved: *“It’s both about balance and the strength of the body, in the legs, that I don’t trust my body. Now, my body acts in a way that I’m not used to…or that I don’t know when my leg suddenly buckles”* (P16). Impaired visual function crucially impacted on balance control: *“My vision affects my balance since...I’m not able to focus my gaze”* (P3). Furthermore, simultaneous head–eye movements caused loss of orientation in space, which impacted balance control: *“To look where someone is pointing ‘hey, look over there’, then I can’t just turn around like that because if I do that then I’m off”* (P2).

Lack of automaticity caused by impaired sensory and motor functions was often compensated for through enhanced attention and concentration. However, the lack of ability to maintain concentration was described as contributing to balance impairment: *“And then I do something automatically without thinking, and it’s gone…I lose my balance, because I have to think about it”* (P6). Some participants had realized that they could no longer focus on other things while moving around and adapted a one-task-at-a-time strategy: *“I can’t talk on the phone while I walk. If someone calls, I stop (to answer) because I lack stability and control”* (P10), or *“It feels like I can’t think about two things at the same time* (P16).

More complex, stimuli-rich, environmental conditions (such as being in a crowd) could largely impact on the ability to keep one’s balance. One participant described this as follows: *“When the surrounding environment is shifting rapidly, I lose focus and fall”* (P10), and concretized the concept further: *“It’s difficult to walk in a crowd, because the reference points I use move. The crowd is moving in different directions”* (P10). Furthermore, unstable ground conditions influenced balance and required attention and concentration: *“I constantly need to be careful and think about what I’m doing when I’m on a boat or on a moving surface”* (P14). Another environmental condition that impacted balance control was high temperatures; heat-intolerance contributed to the activation of latent impairments affecting balance, such as weakness, dizziness, or fatigue.

Fatigue, lacking physical and/or mental energy, was perceived as generally affecting balance control; ie, balance deteriorated as fatigue increased. Energy was important for maintaining balance and moving around safely, described as *“My energy runs out and I kind of collapse, like a folding knife”* (P2), or as *“I get so tired that my vision becomes blurred, and I move unsteadily”* (P10). Stress, either originating within the individual or from their surroundings, also affected balance: *“Stress is destructive to my balance; both my own stress and if someone around me is stressed”* (P3). Beside affecting balance, being stressed contributed to fatigue, which was perceived as having a blocking effect on movement: *“If I’m stressed, it’s almost like I’m moving more slowly than otherwise…If I feel like oh, I must get to the subway or the bus before it departs, it’s like my body automatically pulls the brake”* (P12).

### Burdens of Balance Impairment

Deteriorated balance control brought changes into participants’ daily routines, experienced as burdens negatively affecting one’s lifestyle. This constrained spontaneity in activities that required balance control, such as activities in daily living, work, leisure activities, and physical exercise, as 1 participant expressed: *“Without the balance impairment, everything would be easier in my everyday life. I could be more spontaneous”* (P10). Impaired balance also restricted social participation: *“It’s boring not being able to join in when you meet new people, who invite you to, and who take the initiative to do things”* (P13). Furthermore, striving for autonomy by avoiding burdening others for help with maintaining balance control restricted participation: *“If they say: ‘Are you coming? We will have lunch over there’. I say: ‘No, it’s too far away’. Although I could join them if I had had some support for my balance”* (P5).

Among those experiencing fatigue, balance impairment was described as an additional burden, a barrier, and a frustration, further hindering performance in activities. One participant described the impact of fatigue on balance, movement, and activity: *“Yes, I feel a little…sort of fragile. If I had the energy, I know I would be able to. But it’s this bloody energy”* (P2). Unpredictable day-to-day variation in physical and/or mental capacity contributed to insecurity and distrust when moving around: “*Having an affected balance means that you become less secure with everything that actually is just as usual, but now you perceive it differently*” (P6). Trust, to be secure in one’s own capacity in the actual environmental conditions, was described as necessary to increase one’s confidence and to keep balance: *“But the feeling of having something to hold on to, whether it’s needed or not, makes me feel more secure”* (P15).

Worsening of balance control led to grief and concerns as expressed in the following quote: *“My affected balance makes me sad…but now I’m mostly worried since it’s getting worse, and it’s not manageable anymore…it feels worthless”* (P16). Furthermore, it affected self-image and identity: *“My self-image is that I’ve been quite, or I still am, quite independent…I’ve been physically capable of managing challenging situations…and now, suddenly...like with this disease in general...my self-image has changed to something slightly different”* (P4).

Impaired balance also led to falls, described as an unpredictable and sometimes stressful event: *“I don’t know when my leg may suddenly buckle, if I’m standing and doing something”* (P16). Falling was described as an unforeseen event, which could be violent, as 1 participant described: *“It feels like someone grabs me and just throws me to the ground”* (P3). Participants linked falls both to impairments and to environmental conditions: *“Well, I’ve fallen. I stumble. It can be something small, tiny, a pebble stone or something...my foot bends and I suddenly have no...I have no balance. I fall like a…it’s like falling flat. I can’t do anything*” (P8).

### Management of Balance Impairment

To manage and come to terms with current disabilities was an ongoing time-requiring processing of psychologically based strategies related to coping, described as: *“I don’t want that, I don’t want it to look like I’m ill”* (P12). There was an ambiguous feeling to being dependent: *“I need others to help me, and I don’t like that”* (P12). Grief over the disease progression was, in turn, considered by some to be a waste of energy: *“It’s not worth worrying about things, it’s just a waste of energy”* (P15).

Managing the balance impairment and the risk of falling required attention and awareness. Often, it also had to be simultaneously dealt with, together with other people’s attitudes, comments, or worries; as 1 participant said: *“I’ve tripped in the subway several times, and there’s such a commotion when it happens. Falling in the subway gets too much attention”* (P10). Participants perceived this as not only trying to be in control of one’s own balance, but also being responsible for others’ reactions, which for some led to further avoidance of being active. However, sometimes reasoning with others was positive and contributed to choices about how to consider balance impairment in various activities.

One management approach was acceptance of disability, exemplified by: *“I have to relate to it and accept it. Because otherwise I’ll become a victim, with self-pity, and that’s not who I am”* (P9). A struggle to accept being less ambitious to better manage valued activities could emerge: *“I have to be more careful with what I do and how I plan to do things. Because in my head I want to do a lot more than I’m capable of”* (P2). Being unfamiliar with current disabilities, their progression and how it could influence balance control contributed to feelings of -not having control *“I feel that I simply can’t control my own body, the way I’d like to. Everything happens approximately, my movements become more and more approximate”* (P6). Another participant described: *“I think I’m lifting my foot, but apparently, I’m not”* (P2).

Strategies for managing balance impairment included stress management by planning, prioritizing, and doing one thing at a time, exemplified by: *“I avoid all stressful situations, make sure to be on time, and I really try to sort through what I should do”* (P14). The strategy of planning was a necessity that enabled spontaneity in chosen activities: *“To better administer my balance, I could use the bike as a walking aid…it doesn’t mean that I can’t be spontaneous, but it just means that if I bring my bike, I can be spontaneous without problems”* (P4). Further, compensatory actions, aiming at maintaining balance control, were the bridging through substitute functions to counteract balance impairments. The use of visual function was an important compensatory action: *“I have to watch where I put my feet”* (P11) and *“With my sight I can see if I’m about to fall, and then I can save myself. Truly, vision is a necessity”* (P5).

Experiencing fatigue led to constantly being preoccupied with energy management so to not overextend oneself: *“I always try to have control over how much capacity I have left, but at the same time I don’t want to have too low a level of ambition. Because, I want to endure, so to speak. But, if I push myself too hard, the next day will be completely wasted”* (P10). Recovery routines to generate energy were described as vital for maintaining balance control throughout the day. Finally, management of impaired balance through communication about ones’ difficulties could be a beneficial strategy, especially communication around the use of walking aids: *“When I use a crutch. Then, gosh, everyone is incredibly kind. They help me, move to the sides, and hold up doors, and... they’re all very kind”* (P5).

### Negotiation Between Capacity and Ambition for Continuing the “Good Life”

Impaired balance control impacted the ability to perform activities that were highlighted as important and valuable in living a good life. The reasoning about choosing whether to perform valued activities was sometimes made by the individual themselves and was sometimes made together with others, eg, a next of kin or a health care professional. However, sometimes performing these activities was described as “worth it” even if it resulted in fatigue, concerns of falling, and associated consequences, as highlighted in the following: *“Concerns about the consequences of falling doesn’t stop me from carrying on, I still expose myself to things anyway. Otherwise, life wouldn’t be fun either”* (P16). To not stop living by still choosing an active life was also highlighted from another perspective: *“After all, I don’t stop living. It would be sad. Because I’ve ceased to do so many things, that I must keep on with some of the fun things (ie, activities) too. Even if I don’t have the energy”* (P10).

Impact on quality of life by activity limitations, often originated in a lack of energy, as 1 participant said: *“It’s not such a fun life. It’s pretty boring...Simply, my fatigue affects me”* (P12). Being limited but still in charge of how one’s resources should be used was described as valuable. It was expressed that one’s mindset was important to have a quality of life despite one’s limitations, as 1 participant said: *“I still have a good life”* (P14).

## Discussion

This qualitative study had a novel approach by exploring PwMS’ experiences of living with impaired balance control and how it impacts activity and can be managed in everyday life. Maintaining balance control—which used to be an automatic skill required in various activities—is now needed to be dealt with consciously. The balance deficiencies limited and affected performance and participation in everyday activities. Furthermore, what one did or intended to do before, during, and after an activity had to be thoroughly considered, since the ability to maintain balance and be continuously active was often limited due to a lack of energy and to day-to-day variation in capacity. Independence in various activities was highly appraised, but remaining independent during activities requiring balance control was often perceived as too energy demanding and costly. However, to keep up a good life, it was sometimes worth performing valued activities that threatened one’s balance, no matter what risk it entailed.

The diverse individual perceptions of how the occurrence of impairments affected balance control, as described in this study, highlights the need for different approaches for its management in PwMS. For many, physical impairments were the starting point for describing factors impacting balance, whereas aspects of cognitive function, such as attention and concentration were often described as either needed to compensate for physical shortcomings or being in short supply itself. Additionally, the consequences of fatigue on balance performance were accentuated. In this study, the complexity of how various physical and cognitive shortcomings affect balance and need to be adjusted in consideration of daily capacity and energy levels was highlighted. This is in line with recent results describing the large impact of MS-related fatigue on everyday activities,^28^ where the need of constant adjustments to one’s present energy level is highlighted. This emphasizes the need for balance interventions to counteract balance impairment and to support and strengthen the individual with MS to find strategies to remain active. In such an intervention, the uncertainties concerning unpredictable day-to-day variation in capacity need to be reflected upon. The shortcomings described as influencing balance control in PwMS (sensory, motor, and cognitive functions) are in accordance with Horak recognized model for balance control.^3^ However, our results imply that fatigue and unpredictable day-to-day variation in capacity are prominent aspects for balance impairment in PwMS, which need to be considered in an MS-adapted version of Horak model.

Some participants described that the environment could affect their balance when being active.^29^ Difficulties in focusing on keeping one’s balance, and at the same time registering, interpreting, and reacting to ongoing environmental stimuli were highlighted. This contributed to increased fall risk and activity curtailment. Experiences of how external stimuli influenced their balance helped participants in reducing its impact. Behavioral aspects contributing to the minimization of environmental impacts are crucial; however, there is still limited evidence as to how PwMS should be supported to change such behavior and thereby manage the impact of environmental stimuli themselves.^30^ Moreover, the complex conditions described as affecting balance control highlight the need for each PwMS to explore and understand how they interact and impact the performance of activities. This knowledge is often difficult to acquire alone, therefore counseling organized by health care providers is needed and should be offered. Such counseling might reveal functional impairments and disease progression, which can be an unwanted and painful discovery for the patient. Therefore, this communication must be personalized and delivered with sensitivity.^31^

Aminian and colleagues,^20^ have shown how balance impairment, risk of falling, and the attitudes of other people are aspects contributing to activity limitations. Other studies have reported that mobility problems, motor and visual impairments, and fatigue, affect social participation and that decreased social participation is associated with depression^32^ and low quality of life.^33^ These results are in line with findings in the present study. Beside the impact on activities and participation, impaired balance control influenced identity and self-image and reduced one’s confidence in their own capability. Such negative thoughts might create barriers to resilience and management of disease progression.^34^ To oppose such thoughts, participants used psychologically and physically based coping strategies, which contributed to resilience by not allowing balance impairment to greatly impact their lives. Furthermore, deciding for oneself whether or not to continue doing valued activities—even if they could result in falling or becoming fatigued—was expressed as important for an individual in the pursuit of the desired life. The process of discussing pros and cons of one’s choices with others was described as contributing to a better self-image. These results highlight the importance for health care to, when needed, support patients in their autonomous decision-making process, a support recently highlighted as necessary in order to remove barriers to improved quality of life in PwMS.^35^

### Methodological Considerations

To achieve trustworthiness^36^ in this study, a checklist to improve trustworthiness^37^ was used. The study sample was strategically selected, and the mini-BESTest was used to ensure that the sample experienced impaired balance control. The results are therefore transferable to similar groups of PwMS but not to those with very different characteristics, or to other patient groups.

For consideration of the data’s dependability, participants were interviewed at least 2 months after participation in the reliability study, so that the statements and descriptions would not directly link to previous participation. Furthermore, the interviewer had not been involved in data collection in the reliability study.

Themes and formulation of open-ended questions in the interview guide were based on previous studies and on the authors’ clinical experiences of meeting PwMS with impaired balance. Having physical therapist experience working with PwMS might contribute to preconceptions and biases. However, it can also serve as valuable keys to reaching deeper meaning in the interviews.

To ensure authenticity during the analysis process, original interviews were reviewed iteratively. All interviews were initially analyzed by the first author, and to guarantee similarity between interviews, each analysis step in each interview was discussed between the first and the last author. To increase analysis transparency, quotations that best mirrored the content of each category were selected when reporting the results.

### Clinical Implications

This study highlights the importance of person-centered health care, which should include not only a standard examination but also the acquisition of information on the individual’s experiences of how impaired balance diminishes participation in various activities. Clinical awareness of the patient’s perspective of balance impairment might increase both the quality and efficiency of physical therapy, and it might, with greater precision, end up in line with the patient’s thoughts of a life in which participation in valued activities is less restricted and/or avoided. Clinical interventions aimed at improving balance control and/or in combination with psychological interventions focusing on related behavioral aspects would be helpful for PwMS to continue being active. This might also provide favorable conditions for increased individual motivation for participation in therapy.

### Limitations

Participants’ prior interest in participating in research on balance control may have influenced the study sample selection since they were recruited from a reliability study of gait and balance assessments. Furthermore, these assessments may have affected participants’ perceptions of their balance control at the time of the interview. This could have been avoided if the interviews had been conducted before participation in the reliability study; but, in that case, participants could not have been strategically selected in regard to their balance performance.

### Conclusion

People with mild and moderate MS disability experienced that their balance control, which had previously been an automatic skill when moving around, now required attention and awareness. Aspects of body functions highlighted as central to keeping balance were somatosensory-motor functions, the importance of vision, and managing mental and/or physical fatigue. Day-to-day variation in capacity and being in stimuli-rich environments were conditions highlighted as impacting balance. Balance impairment was described as having adverse impacts on activities and participation in everyday life, but at the same time, a strong effort was shown to not let these limitations control and determine their lives. To manage the activity limitations and participation restrictions and to move forward in the struggle to keep up a good life, an extensive toolbox of specific strategies aiming to minimize the impact of balance impairment was used to maintain quality of life, despite one’s disabilities.

## Data Availability

The data supporting the findings of this study are available from the corresponding author upon reasonable request.
